# Crohn’s Disease Complicated by Rare Types of Intestinal Obstruction: Two Case Reports

**DOI:** 10.3389/fmed.2022.895202

**Published:** 2022-04-28

**Authors:** Kai Xia, Renyuan Gao, Xiaocai Wu, Yu Ruan, Jian Wan, Tianqi Wu, Fangtao Wang, Yin Lin, Lu Yin, Chunqiu Chen

**Affiliations:** ^1^Diagnostic and Treatment Center for Refractory Diseases of Abdomen Surgery, Shanghai Tenth People’s Hospital, Tongji University School of Medicine, Shanghai, China; ^2^Surgery and Anesthesia Center, Shanghai Tenth People’s Hospital, Tongji University School of Medicine, Shanghai, China

**Keywords:** Crohn’s disease, intestinal obstruction, capsule endoscopy, internal hernia, minimally invasive surgery

## Abstract

Intestinal obstruction is one of the most common complications of Crohn’s disease (CD), jeopardizing the quality of life of patients. Numerous factors may contribute to intestinal obstruction in CD. Thus far, the primary reason has been identified as intestinal fibrosis caused by repeated chronic inflammation during the active phase of CD. Herein, we report two rare complicated CD cases and provide a reference for the clinical diagnosis and treatment of similar patients. Case one involves capsule endoscope retention in the small intestine of one CD patient concurrent with intestinal obstruction. Case two is a CD patient with intestinal obstruction caused by a mesangial hernia and ileal stenosis. Individualized and minimally invasive surgical intervention ultimately resulted in the successful management of these two patients. The two cases serve as an excellent guide for diagnosing and treating CD patients who present with similar symptoms.

## Introduction

Crohn’s disease (CD) is a chronic inflammatory bowel disease that is complicated by genetic susceptibility and environmental triggers (1, 2). It is most frequently diagnosed in individuals between the ages of 20 and 30 (3). At present, the underlying cause of CD is still unknown, and its prevalence is gradually increasing worldwide (4). CD is prone to various complications, with intestinal obstruction being one of the most common. Notably, Lin et al. (5) showed that around 70% of patients inevitably develop fibrosis-associated intestinal stricture 10 years following CD diagnosis, which may contribute to intestinal obstruction. In this study, we described two cases of CD complicated by particular types of intestinal obstruction. The Ethics Committee of Shanghai Tenth People’s Hospital approved this case report (Ethical approval number: SHSY-IEC-5.0/22K33), and informed consent was obtained from all patients.

## Case Report

### Case 1

**FIGURE 1:**
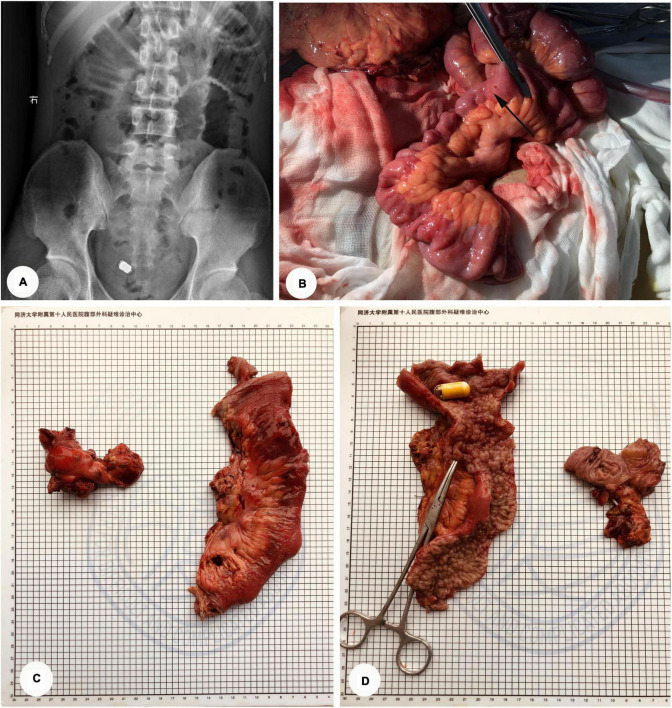
CD complicated by small intestinal obstruction with capsule endoscopic incarceration (Case 1). **(A)** Abdominal plain film: the jejunum in the upper left abdomen was dilated, and the intestinal cavity contained a gas-liquid plane. Metallic shadows could be seen in the overlapping area of the pelvic cavity. **(B)** Intraoperative exploration: the capsule endoscope is incarcerated in the narrowed proximal ileum (Arrow point). **(C,D)** The specimen of surgery: the yellow cylinder is capsule endoscope; vascular clamp showing internal fistula.

On January 25, 2021, a 26-year-old man was diagnosed with incomplete intestinal obstruction in another hospital after experiencing vomiting, abdominal pain, and abdominal distension following a meal. The symptoms improved after fasting, gastrointestinal decompression, anti-infective treatment, and parenteral nutrition support but remained intractable. On March 11 of the same year, the patient experienced severe abdominal pain while undergoing capsule endoscopy in the same hospital, and the capsule endoscope was not excreted after the examination. Enhanced computed tomography (CT) scans of the abdomen revealed small intestinal obstruction, with multiple thickening and strengthening of the small intestinal wall. The obstruction point was located in the small intestine of the pelvic region. According to the images, the first consideration was a CD of the small intestine. As a result, the patient was admitted to the Diagnostic and Treatment Center for Refractory Abdominal Diseases for additional treatment. Physical examination revealed tenderness in the lower abdomen and no other positive signs. The plain abdominal film revealed that the jejunum in the upper left abdomen was dilated, and the intestinal cavity contained a gas-liquid plane. Moreover, metallic shadows could be seen in the overlapping area of the pelvic cavity (Figure 1A). The patient was diagnosed as “incomplete small intestinal obstruction, CD (?), small intestinal foreign body (capsule endoscope).” The patient then underwent surgery to remove the thickened intestine and foreign body on March 19. During the operation, laparoscopic exploration revealed that the small intestine presented a segmental lesion intertwined with stenosis and dilation. After exploring the entire small intestine from the Treitz ligament, we discovered that the narrowest point of the intestine was approximately 180 cm from the Treitz ligament, and the stenosis segment of the intestine was approximately 30 cm in length. The distal small intestine was almost normal, while the terminal ileum was narrowed and thickened. Endo-GI was used to resect the intestinal cavity at the beginning of ascending colon, dragging out the narrowed ileum, capsule endoscope incarcerated in the proximal ileum was touched (Figure 1B). Then segmental resection of the small intestine with side-to-side stapled anastomosis was performed. Finally, the normal terminal ileum was severed, and the terminal ileostomy was completed. An ileus tube was inserted from the nose to the duodenum. The entire operation lasted 2 h, and the blood loss during the process was about 100 ml. The surgical specimen was inspected, and the multiple polyps of the intestinal wall and capsule endoscope were removed (Figures 1C,D). The patient had an uneventful postoperative period and was discharged 8 days after surgery. Pathology analysis revealed that multiple polyps were seen on the mucosal surface of the small intestine with a paving stone-like appearance, the diameters of which were about 0.2–0.6 cm. A longitudinal ulcer was seen on one side of the surgical margin, with a length and width of approximately 4 and 0.9 cm, respectively. Microscopy revealed acute and chronic intestinal wall inflammation with plasma cells, neutrophils, and lymphocytes infiltration. The ulcerative area reached the muscle layer and was alternated with inflammatory polyps. Combined with clinical history, the diagnosis of CD was considered. The patient underwent ileostomy closure surgery after 3 months. The patient is currently in good condition, the disease is well controlled, and no apparent complications have been reported.

### Case 2

**FIGURE 2:**
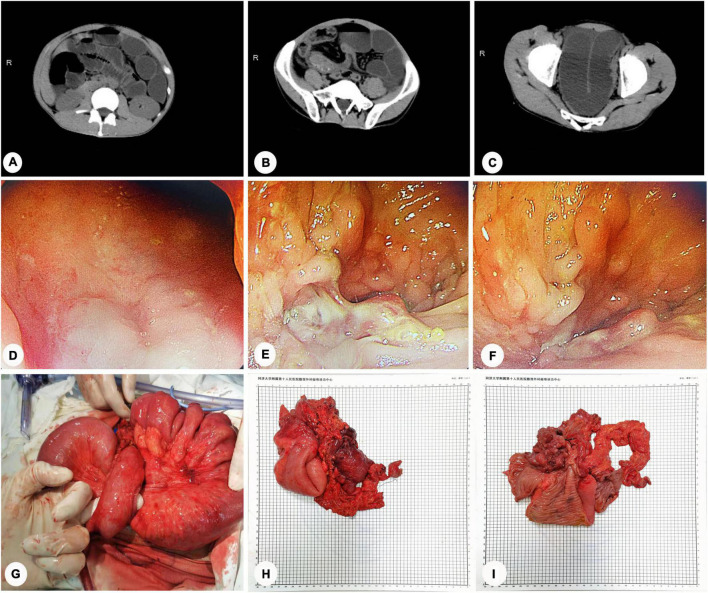
Intestinal obstruction caused by CD combined with internal hernia and ileal stenosis (Case 2). **(A–C)** CT scans of abdomen: intestinal tract lesions in the right middle and lower abdomen, causing a proximal small intestinal obstruction. **(D–F)** Endoscopic examination: noticeable mucosal swelling, irregular fissure ulcers, and multiple polypoid hyperplasias in the sigmoid colon (18 cm away from the anus). The intestinal cavity was so narrowed that the endoscope could not pass through it. **(G)** Intraoperative exploration: the proximal small intestine passed through the transverse mesocolon, causing the formation of the internal hernia. **(H–I)** The specimen of surgery.

In 2009, a 36-year-old man with lower abdominal pain and palpable right lower abdominal mass was diagnosed with CD. Since then, he has been followed up regularly in our Gastroenterology Department and treated with medication to slow the progression of the disease. Four days prior to admission, the patient experienced severe abdominal pain and distension following a full meal, which was accompanied by four to five episodes of vomiting. As a result, the patient was admitted to our department for follow-up care. Further examination of the patient’s medical history revealed that the patient had not had any excretion or exhaust in the preceding 3 days and had previously defecated two to three times per day. Physical examination revealed tenderness in the middle and lower abdomen, with a palpable mass in the lower abdomen. CT scans of the abdomen showed intestinal tract lesions in the right middle and lower abdomen, causing a proximal small intestinal obstruction (Figures 2A–C). Endoscopic examination indicated noticeable mucosal swelling, irregular fissure ulcers, and multiple polypoid hyperplasias in the sigmoid colon (18 cm away from the anus). The intestinal cavity was so narrowed that the endoscope could not pass through it (Figures 2D–F). As a result, the patient was diagnosed as “ CD (A2 L2 B2 P, active phase), small intestinal obstruction, sigmoid stenosis, severe malnutrition (BMI 16.41 kg/m^2^).” After admission, a transnasal ileus tube was inserted under interventional guidance. 1,200, 200, 100 and 10 ml of dark green liquid per day were drained from the tube 4 days before the operation. The patient felt no more abdominal pain after that intervention. Following strict evaluation, the patient underwent subtotal colectomy, ileocecal resection, laparoscopic intestinal adhesiolysis, plication of small intestine, and ileostomy on November 30, 2021.

During the operation, laparoscopic exploration revealed that the proximal small intestine was significantly dilated. Furthermore, a mass wrapped by the omentum majus was seen in the ileocecal junction adhering to the lateral abdominal wall. When an ultrasonic scalpel separated the ileocecal mass, pus was seen flowing from the mass. We then switched to laparotomy because of the unclear anatomical structure and limited vision during the laparoscopic operation. During exploratory laparotomy, contractures were found in the ascending, transverse, descending colon, and mesangium. The proximal small intestine passed through the transverse mesocolon, causing the formation of an internal hernia (Figure 2G). Then, a linear cutter stapler severed the descending colon sigmoid junction, and the ileus tube was delivered from the proximal ileum to the terminal ileum. After the ileocecal mass was dragged out, the specimen was severed with a linear cutter stapler at a distance of 10 cm from the ileocecal junction. The terminal ileostomy was then completed. The entire operation lasted 4 h, blood loss during the process was about 600 ml, and the length of the residual small intestine was about 200 cm. The surgical specimen was inspected, as shown in Figures 2H,I. The patient had an uneventful postoperative period and was discharged 10 days after surgery. Pathology analysis revealed a protrusion lesion in the ileocecal area, measuring 10 × 4 × 4 cm. Under the microscope, chronic active inflammation of colonic mucosa could be seen, with erosion, fissure ulcer and polypoid changes. Lymphoplasmacytic cells were distributed in the colonic mucosa focally. Combining his past clinical history, the diagnosis of CD complicated by intestinal obstruction was given. Since the postoperative follow-up, the patient is in good condition, the disease is under control, and no apparent complications have been reported.

## Discussion

In CD patients, intestinal obstruction resulting from capsule endoscopy is uncommon. However, according to Guevara-Morales and Castellanos-Juárez (6), the capsule endoscope retention rate in CD patients could reach around 5–6%. Therefore, clinicians should pay closer attention, as this can significantly impact these patients. Capsule endoscopy is a minimally invasive procedure that enables visualization of the entire small intestine mucosa in patients with known or suspected CD (7). The widespread use of capsule endoscopy has significantly compensated for the shortcomings of colonoscopy and enteroscopy. However, intestinal obstruction is recognized as one of the most common complications of CD, which increases the possibility of retention of the capsule endoscope in the gut (8). Therefore, it is necessary to evaluate intestinal cavity patency before swallowing capsule endoscopes. Rozendorn et al. (9) revealed that the ability of magnetic resonance enterography (MRE) to predict capsule endoscope retention in patients with CD is relatively significant. The sensitivity and specificity for the predictions by two radiologists were 92.3, 59% and 100, 52.3%, respectively. Minordi et al. (10) further uncovered that MRE provides a superior soft-tissue contrast resolution and a better visualization of the intestinal wall and its inflammatory and fibrotic characteristics. In addition, computed tomography enterography (CTE) is another well-established diagnostic technique for evaluating the intestinal cavity’s patency. Therefore, capsule endoscopy should be used with caution in patients with CD complicated by bowel stenosis.

In case 2, the primary cause of intestinal obstruction is the formation of trans-mesenteric internal hernia. According to the literature, the incidence of internal hernia is about 0.2∼0.9%, accounting for 0.6∼5.8% of all intestinal obstruction cases (11). Internal hernia frequently exhibits no characteristic clinical manifestations prior to the formation of intestinal obstruction, resulting in a low rate of diagnosis preoperatively. Moreover, closed intestinal loops can form quite easily, resulting in intestinal strangulation and necrosis. Patel et al. (12) concluded that when an internal hernia is complicated with acute intestinal obstruction, the mortality rate can reach up to 50%. Therefore, early detection and surgical intervention are critical for optimizing disease outcomes.

Medical treatment is frequently ineffective in CD patients who developed intestinal obstruction, and in both cases, the obstruction was eventually resolved through surgical intervention. Endoscopic intervention or surgical resection is primarily used to treat intestinal stenosis in CD. Endoscopic balloon dilation (EBD) is indicated when the length of strictures is ≤ 5 cm, non-angulated, with a sizeable intestinal cavity large enough to allow balloon dilators in the absence of contraindications such as the presence of fistula, abscess, or malignancy (13, 14). In addition, endoscopic stricturotomy (ES) is also an excellent choice when strictures are non-angulated, and the length of the narrowed intestinal cavity is short. Nan et al. (15) demonstrated that the reoperation rate following ES is significantly lower than that following EBD and that ES can result in a higher surgery-free survival rate. However, surgical resection is required when the length of the narrowed intestinal cavity is excessively long or when endoscopic therapy is contraindicated (16). Our department has treated a large number of patients with CD and has accumulated much surgical experience. The narrowed bowel was severed in both cases, and the small intestine cavity was also dilated using an ileus tube. The small intestine must be plicated when the segments of narrowed bowel are long, and the length of the small intestine is insufficient for intestine resection. Not only can plication of the small intestine be used to dilate the intestinal cavity, but it can also be used to prevent the development of adhesive ileus following surgery.

Notably, our department emphasizes minimally invasive surgery, putting the concept of enhanced recovery after surgery (ERAS) into practice. By minimizing pain, hospital stay, and adhesion formation, minimally invasive surgical options have significantly reduced postoperative morbidity (17). Our team recently published a retrospective study confirming the safety and efficacy of laparoscopic minimally invasive surgery as a viable alternative therapeutic option for patients with CD (18).

In conclusion, there are numerous CD cases complicated by intestinal obstruction, and surgery is the mainstay modality to relieve the obstruction. As surgeons, we should thus choose individualized and minimally invasive options that will result in long-term benefits for CD patients.

## Data Availability Statement

The original contributions presented in the study are included in the article/supplementary material, further inquiries can be directed to the corresponding author/s.

## Ethics Statement

The studies involving human participants were reviewed and approved by the Ethics Committee of Shanghai Tenth People’s Hospital. The patients/participants provided their written informed consent to participate in this study. Written informed consent was obtained from the individuals for the publication of any potentially identifiable images or data.

## Author Contributions

KX: disease diagnosis and treatment, collection of cases, and draft manuscript. RG, XW, and YR: disease diagnosis and treatment, collection of cases, and revise manuscript. JW, TW, FW, YL, LY, and CC: disease diagnosis and treatment, and revise manuscript. All authors contributed to the article and approved the submitted version.

## Conflict of Interest

The authors declare that the research was conducted in the absence of any commercial or financial relationships that could be construed as a potential conflict of interest.

## Publisher’s Note

All claims expressed in this article are solely those of the authors and do not necessarily represent those of their affiliated organizations, or those of the publisher, the editors and the reviewers. Any product that may be evaluated in this article, or claim that may be made by its manufacturer, is not guaranteed or endorsed by the publisher.
